# One-and-a-Penny-Ha'Penny

**Published:** 1895-08-31

**Authors:** 


### ONE-AND-A-PENNY-HA'PENNY.

In the reign of George the Third, the Government being pressed for money, invented a new tax. They passed an act "to grant to His Majesty a stamp duty on licences to be taken out by certain persons uttering and vending medicines, and certain stamp duties on medicines sold under such licences, or under authority of His Majesty's Letters Patent." Those persons were to take out these licences who possessed or claimed to possess "any occult, or secret, or unknown art, or some exclusive right or title to the manufacture of specifics or otherwise, for the cure or relief of any ailment or malady incident to, or in any way affecting, the human body." This act, meant only as a source of revenue, or perhaps intended to restrain the selling of drugs by the ignorant, has resulted in giving a prestige to patent medicines, which largely increases their sale. It is not the only time that Acts of Parliament, intended to restrict an evil, have created a monopoly; the licensing of public-houses was intended to lessen drunkenness, and one of its most prominent results has been to raise the value of "licensed houses" enormously, and to make the publican an important citizen. The result of the stamp duty on patent medicines has been to give the public the impression that the Government has some knowledge of the mysterious and often nauseous compounds they accept so meekly, and to which they have given the seal of their approval.

It would be a great blessing if some responsible analyst were appointed to investigate the components of every drug that claimed a licence, and were to judge by its merits whether it deserved any special privilege beyond insertion in the "British Pharmacopœia." But if this were done the stamp duty would go down wofully. It is doubtful if enough would be left to pay the analyst's salary. The makers and vendors of patent medicines cling to the permission to keep their art "occult, secret, and unknown," and avow to none the simple fact that their tonics are chiefly made of aloes, their soothing syrups of opium, and their electricities of distilled water. Of course, if one were to put it direct to Mother Seigel or Count Mattei they would probably own to the aloes and the water — Mrs. Winslow would stoutly deny the opium for fear of avenging mothers—but they would add that in their compounds there was something else—something which, as some pretend it is beyond the power of chemical analysis to discover. What is that something, then? Aye, there's the rub! For Count Mattei and Mother Seigel might justly say, "If we publish the name of this component every druggist in the kingdom can make our mixtures as well as we ourselves, and we lose all the profit of our research and ingenuity." Now, it is fair enough that an inventor or discoverer should be the chief gainer by his own thought and skill, and it is to secure this that our patent laws are made; but every one who takes out a patent is compelled to deposit a description of it; only the vendor of patent medicines is allowed to obtain what is, in the eyes of the general public, a Government guarantee and monopoly for something whose nature, constituents, and action are totally unknown. Surely it is not too much to ask that someone, chemist or medical man, should be informed wherein lies the powers of the drugs that are sold under the Government stamp—powers which, if one may believe the advertised account of cures, are little short of miraculous.

But the patent medicine men make much of the jealousy of the regular profession. Doctors have no objection to some patent medicines; certain of them are frequently prescribed, usually because the drugs they desire to administer are in these combined in a more attractive way than the local druggist could attain to. The doctors use certain patent medicines, which they have analysed, and from which they have seen good results. They prescribe these, not, indeed, for every ailment under the sun, wherein they differ from the average advertisers of patent panaceas; and they do not prescribe compounds which seem to them useless or even dangerous. On the contrary, they protest against these, and are called jealous for their pains.

Wherein, however, does this jealousy lie? Primarily in a conviction that a man who has studied a subject is likely to know more about it than one who has not. The justice of this contention is not disputed in a general way; but you will always find men who know more about medicine than the doctor, more about law than the solicitor, and more about theology than the parson. They have been known to instruct their cooks, either wife or hired maiden, in the preparation of the dinner; but usually retire worsted from that encounter. It is such as these who spread the fame of patent medicines. In the first place they are probably inaccurate about the ailment they suffered from, and resent any inquiry that seems to minimise its seriousness. In the second, they are inclined to testify to the value of the cure before they have given time to see if it is permanent. Their testimonials sometimes remind one of the converted drunkard who told a revival meeting that he had been a drunkard for ten years, but had been converted the day before and had not touched alcohol for twenty-four hours. It was surely too soon to testify; relapses are not unknown in such a case. And one cannot but note that the testimonials mostly come from uneducated people. "Not many wise are called," and those who know the vague and inaccurate talk about illness that goes on among the ignorant will doubt if the washerwoman whose "life was a burden to her" till she met with Mother Seigel, or the gardener who "could not move without agony" until Sequah had pity on him, are to be credited as fully as could be desired. Not that Mother Seigel and Sequah were wholly useless. The famous syrup is largely composed of aloes, and is sweetened with liquorice, and if the estimable lady had consulted the nearest doctor, it is probable that he might have discovered that she had a torpid liver, which makes life a burden to most folks, and would have prescribed aloes and liquorice, too. But he would not have asked for a testimonial, and he would certainly have given her more for the money. Similarly, fish oil, turpentine, and oil of camphor—the constituents of Sequah's Oil—may, when well rubbed, relieve rheumatism, but they are none the more efficacious for being accompanied by lies and a brass band. We do not deny that these much advertised patent medicines are useful in certain cases—say one out of the score of ailments each undertakes to cure; but they are no more useful than the same drugs labelled by their proper names, and used by the orthodox practitioner. Why, then, should not the public buy its own drugs under either their patent or their ordinary names, and save the doctor's fee? For one very sufficient reason: because the doctor knows, and the public does not, when to administer these drugs, and when to avoid them. If it were only a matter of people wasting their money on medicines, no one need protest, because if forbidden that form of indulgence they will in all probability waste their money in something else; but it is another matter when they injure their health, as many do, by taking medicines which are altogether unsuitable to them. We may dismiss Count Mattei's electricities as harmless, because chemical analysis can find nothing in them but water; but of how many other patent medicines can we even say as much? And yet, because to a shilling's worth (more or less) of water, aloes, paper, print, and puffery, he has added a three half-penny Government stamp, any one in the kingdom may sell patent and dangerous drugs to the lieges without let or hindrance. If we are forbidden to describe our chicory mixture as coffee, if we are not allowed the fond delusion that margarine is dairy butter, why should we yet be permitted to consume our aloes under the name of Prairie Flower?

